# Modulation of the Metal(loid) Specificity of Whole-Cell Bioreporters by Genetic Engineering of ZntR Metal-Binding Loops

**DOI:** 10.4014/jmb.1911.11053

**Published:** 2020-02-10

**Authors:** Hyojin Kim, Geupil Jang, Bong-Gyu Kim, Youngdae Yoon

**Affiliations:** 1 Department of Environmental Health Science, Konkuk University, Seoul 05029, Republic of Korea; 2 School of Biological Sciences and Technology, Chonnam National University, Gwangju 61186, Republic of Korea; 3 Department of Forest Resources, Gyeongnam National University of Science and Technology, Jinju 52725, Republic of Korea

**Keywords:** Heavy metals, protein engineering, whole-cell bioreporter, ZntR, metal-binding loop

## Abstract

Bacterial cell-based biosensors, or whole-cell bioreporters (WCBs), are an alternative tool for the quantification of hazardous materials. Most WCBs share similar working mechanisms. In brief, the recognition of a target by sensing domains induces a biological event, such as changes in protein conformation or gene expression, providing a basis for quantification. WCBs targeting heavy metal(loid)s employ metalloregulators as sensing domains and control the expression of genes in the presence of target metal(loid) ions, but the diversity of targets, specificity, and sensitivity of these WCBs are limited. In this study, we genetically engineered the metal-binding loop (MBL) of ZntR, which controls the *znt*-operon in *Escherichia coli*. In the MBL of ZntR, three Cys sites interact with metal ions. Based on the crystal structure of ZntR, MBL sequences were modified by sitedirected mutagenesis. As a result, the metal-sensing properties of WCBs differed depending on amino acid sequences and the new selectivity to Cr or Pb was observed. Although there is room for improvement, our results support the use of currently available WCBs as a platform to generate new WCBs to target other environmental pollutants including metal(loid)s.

## Introduction

Owing to anthropogenic activities, environmental contamination with various pollutants, including hazardous chemicals, antibiotics, and heavy metals, is accelerating. Since these toxic pollutants threaten human health, effective remediation strategies have become increasingly important. However, such processes are often implemented long after the fact, emphasizing the importance of the early detection of environmental contaminants. Environmental pollutants are generally quantified by using analytical instruments, but instrumental analyses are limited by high costs, time-consuming processes, and technical requirements.

Bacterial cell-based biosensors, referred to as whole-cell bioreporters (WCBs), can overcome the shortcomings of instrumental analysis. Although WCB systems still require optimization, their simplicity and cost-effectiveness have prompted their development and application for the monitoring of environmental pollutants. WCBs function as sensors by the fusion of a promoter that responds to specific stimuli and reporter genes; sensing systems based on various pairs have been reviewed in detail [1-4]. Although targets of WCBs are diverse and various biological events are induced by interactions between sensing domains and targets, the genetic systems for biosensors in bacterial species are limited compared to the number of environmental pollutants. Furthermore, the specificity of sensing domains varies, and they typically respond to multiple targets. Additionally, compensatory pathways make it difficult to improve specificity and selectivity by blocking a single pathway.

We have previously proven that the modulation of the sensing domain changes the selectivity of WCBs and the deletion of genes involved in metal homeostasis enhances sensitivity [5-7]. Since WCBs based on the promoter of the *znt*-operon and *egfp* pair are regulated by ZntR, which interacts with Cd and Hg, this protein was used to quantify the amount of Cd in contaminated soils. When the metal selectivity of ZntR was changed by replacing the metal-binding loop (MBL) region, the specificity of WCBs to Hg increased. Moreover, the detection ranges of WCBs are tunable by replacing reporter genes [8]. Many efforts have been made to improve the properties of WCBs and to modulate the specificity and sensitivity by genetic modifications [9-11].

In this study, we engineered ZntR to generate WCBs that possess novel properties and to demonstrate a rational design process. The amino acid residues of MBLs in ZntR, CCGTAHSSVYC, were mutated one-by-one to generate novel WCBs. Our results show that the promoter and reporter gene pair could provide a platform to generate novel WCBs.

## Materials and Methods

### Materials

*Escherichia coli* DH5α was used as the host strain for WCBs. The Quick & Easy *E. coli* Gene Deletion Kit (Gene Bridges, Germany) was employed to generate mutant *E. coli* DH5α strains. pET-21(a) and pCDF-Duet (Novagen, USA) were used to construct the plasmid carrying the reporter gene with the promoter of the *znt*-operon and carrying *zntR* and its mutants, respectively. Heavy metal(loid)s AsCl_3_, SbCl_2_, CdCl_2_, CrSO_4_, NiCl_2_, HgCl_2_, PbSO_4_, ZnCl_2_, CuSO_4_, and AuCl_3_ were purchased from Sigma-Aldrich (USA) and used as 1 mM stock in distilled water. HotStarTaq and Turbo Pfu were used for amplifying genes and site-directed mutagenesis, respectively, and were purchased from Qiagen (Germany). Restriction enzymes and T4 DNA ligase were purchased from Takara Korea Biomedical (Korea). Primers used in this study were synthesized and purchased from Macrogen (Korea).

### Deletion of Endogenous Genes in *E. coli*

The deletion of genes was performed following a previously described protocol [6]. Briefly, the FRT-flanked PGK-gb2-neo cassette and target gene sequences were amplified by PCR and introduced into *E. coli* harboring the pRedET plasmid carrying the gene encoding recombinase by electroporation at 1,350 V, 10 μF, and 600 Ω using an Eppendorf Electroporator 2510. Eventually, target genes were replaced with the *kan*amycin resistance gene (kan) upon induction with 10% arabinose. The deletion of target genes was further confirmed by PCR. For double-gene deletion mutants, *kan* replacing *zntR* was removed by expressing FLP-recombinase using the 708-FLPe plasmid, and then *copA* was replaced with *kan*. In this study, two mutant strains of *E. coli*, *zntR::kan* and Δ*zntR*/*copA::kan*, were generated and used as hosts for WCBs.

### Plasmid Construction

The plasmid carrying *zntAp::egfp* generated in our previous studies was used as a reporter construct [7]. The *zntR* gene and its mutants encoding ZntR and its derivatives were cloned into the modified pCDF-Duet vector. Since the original vector has the T7 promoter to control gene expression, isopropyl β-D-1-thiogalactopyranoside (IPTG) induction is required in *E. coli* BL21. For simplicity, the T7 promoter was replaced with the P_tac_ promoter for constitutive gene expression in *E. coli*. The *zntR* gene and mutants were inserted into the modified pCDF-Duet with BamHI/XhoI restriction sites. Some of the mutants were generated by 2-step overlap PCR and others were obtained by site-directed mutagenesis with Turbo Pfu. All mutants generated in this study are listed in Table 1 with the amino acid sequences of MBLs.

### ZntR Recovery Test

*E. coli* DH5α-*zntR* was used as a host strain to generate WCBs harboring pZnt-eGFP only and both pZnt-eGFP and modified pCDF-Duet-ZntR. To verify the role of ZntR and constitutive expression of ZntR under the P_tac_ promoter, two WCBs were tested under identical experimental conditions. To test sensitivity toward heavy metal(loid) ions, previously described procedures were used [7], except for the addition of IPTG. Since WCB based on *zntAp::egfp* regulated by ZntR showed selectivity toward cadmium ions, both WCBs were exposed to 5 μM Cd. Fluorescence was measured using an FC-2 fluorescence photometer after 1.5 h of exposure, and the fluorescence intensity was converted to an induction coefficient defined as the ratio of the eGFP intensity from exposure to metal(loid)s to eGFP intensity without exposure. All experiments were replicated at least 3 times.

### Whole-Cell Bioreporter Assay for Metal(loid) Selectivity

WCBs were generated by introducing plasmid pairs carrying *zntR* mutants and *zntAp::egfp* into *E. coli* DH5α-*zntR*, -*zntR*/*zntA*, and -*zntR*/*copA*. To evaluate metal(loid) selectivity, WCBs were grown in LB broth overnight and then the cultures were transferred to fresh LB and grown at 37°C until the optical density at 600 nm (OD_600_) reached around 0.4. Then, 5-ml aliquots were added to test tubes and a metal(loid) solution (5 μM) prepared as a 1 mM stock solution of AsCl_3_, SbCl_2_, CdCl_2_, CrSO_4_, NiCl_2_, HgCl_2_, PbSO_4_, ZnCl_2_, CuSO_4_, or AuCl_3_ was added for exposure. After 1.5 h of incubation, *E. coli* cells were harvested, and the cell pellet was resuspended in Tris buffer (50 mM Tris-HCl, 160 mM KCl, pH 7.4) before measuring the intensity of eGFP. All experiments were replicated at least 3 times.

### Fluorescence Measurements

The fluorescence of eGFP induced by metal(loid) treatment was measured using an FS-2 fluorescence spectrophotometer (Scinco, Korea). Since LB broth interferes with data collection, it was replaced with 50 mM Tris-HCl (pH 7.4) by centrifugation and resuspension. Excitation/emission wavelengths of 470/510 nm were used for measurements, with a 5-nm bandwidth filter. The fluorescent intensity induced by metal(loid) treatment was determined by the induction coefficient defined as [fluorescence intensity of heavy metal(loid)-exposed WCB]/[fluorescence intensity of unexposed WCB].

### Data Analysis

The Dunnett Program (Ver 1.5) was used for statistical analysis of all data when the minimum difference between the control and the treatment means was statistically significant. The analysis was performed with a 95%significance level (*p* < 0.05).

## Results

### Rational Design of Metal-Binding Loops

The zinc-binding site of ZntR possesses 3 cysteines on metal-binding loops and 1 cysteine on another location, and they are known to play a pivotal role for interacting with cadmium, mercury, and zinc ions [7, 8, 12, 13]. Since cysteines in the MBL have this role in interactions with metal ions, cysteine residues were targeted for mutation to modulate the metal-binding properties of ZntR. Among 4 cysteines involved in metal ion binding in ZntR, 3 cysteines in the MBL were changed and Cys79 (outside of the MBL region) was maintained. Diverse combinations of amino acid sequences for MBLs of ZntR derivatives were generated and are summarized in Table 1.

To change the metal ion selectivity and specificity, the MBL of ZntR, i.e., amino acid residues 114 to 125 (CCGTAHSSVYCS), was targeted for mutagenesis. From ZntR-WT, 23 ZntR mutants were generated by 2-step overlap PCR and site-directed mutagenesis. First, ZntR-HJ1 was obtained by replacing His119 with Arg because it plays a critical role in PbrR691 to interact with Pb [14]. Then, HJ3 and HJ4 were obtained by additional mutations from Ser124 to Val to interfere with the activity of the hydroxyl group of Ser. Cys115, the second cysteine in the MBL, was altered to Asp, Ser, and Glu to generate ZntR HJ6, HJ7, and HJ8 from HJ4, respectively. The replacement of cysteines with negatively charged residues and hydroxyl groups was expected to change the metal-binding properties. Additionally, Cys114 was changed to Asp, Gly, Ser, and Pro from ZntR WT to generate HJ9–HJ12. Next, ZntR HJ13 was obtained by Ser125Gly mutation and then HJ14 to HJ16 were generated by replacing C115 with Asp, Ser, and Glu on HJ13. HJ17 to HJ20 were obtained by the His119Arg mutation on HJ9 to HJ12. Five additional mutants were generated based on the experimental data for previous mutants. To increase the metal-binding affinity, negatively charged residues, such as Glu and Asp, were inserted into MBL regions to generate ZntR HJ21–25.

### Modulation of Metal-Sensing Properties by Genetic Engineering

**Fig. 1:**
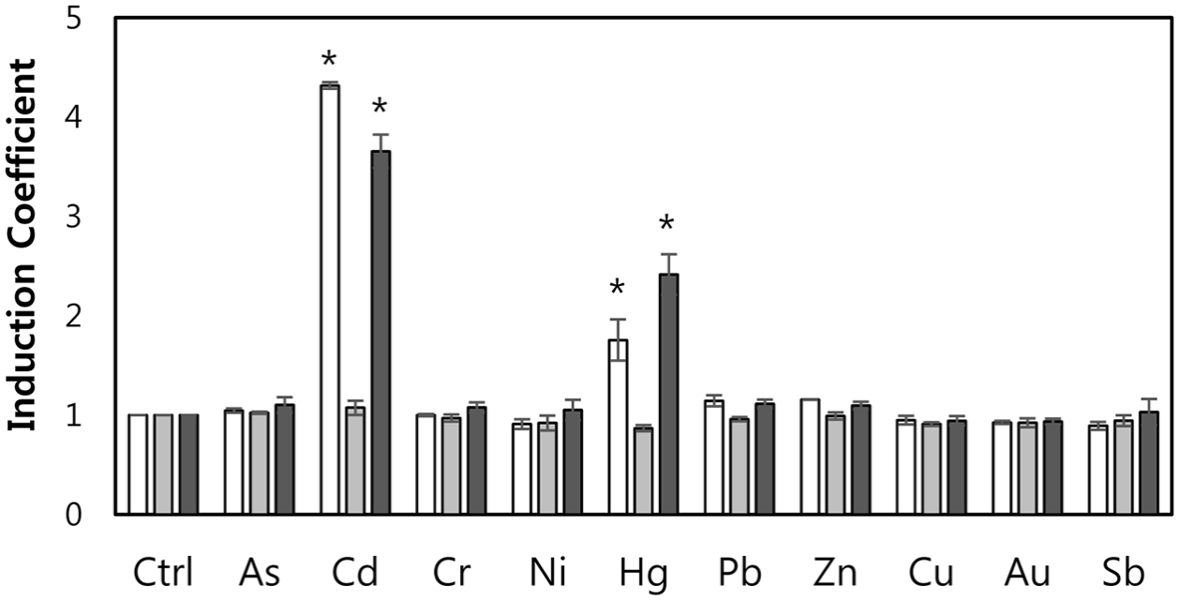
The metal(loid) specificity of WCBs were restored by recombinant ZntR. The induction coefficient values of the WCBs based on *E. coli* WT with pZntA-eGFP (white bar), *E. coli*-*zntR* with pZnt-eGFP (grey), and *E. coli*-*zntR* with pZnt-eGFP/pCDF-ZntR-WT (dark grey) were measured after 1.5 h of exposure to 5 μM of metal(loid) ions. The asterisk (*) means the data were significantly higher than the control (*p* < 0.05).

**Effects of recombinant ZntR.** After generating *E. coli* with a *zntR* deletion, the restoration of ZntR function by inserting endogenous recombinant *zntR* was evaluated to test novel WCBs with alterations in the metal-binding properties of ZntR. The restoration of function has also been verified in our previous study [7]. However, it was necessary to verify the expression of ZntR under the *P_tac_* promoter without IPTG treatment. Three WCB strains generated by inserting pZnt-eGFP into WT *E. coli* DH5α and inserting pZnt-eGFP and pZnt-eGFP/pCDF-ZntR WT into *E. coli* DH5α-*zntR* (*zntR::kan*) were exposed to various metal(loid) ions (Fig. 1). As shown in Fig. 1, WCBs based on WT *E. coli* and the mutant strain with recombinant ZntR showed similar responses toward metal(loid) ions, while WCB without *zntR* showed no fluorescent signals. Although the intensity was weaker for WCBs than for WT *E. coli* DH5α, the metal selectivity of WCBs were not changed, supporting the restoration of the metal-sensing properties of WCBs by recombinant ZntR.

**Fig. 2:**
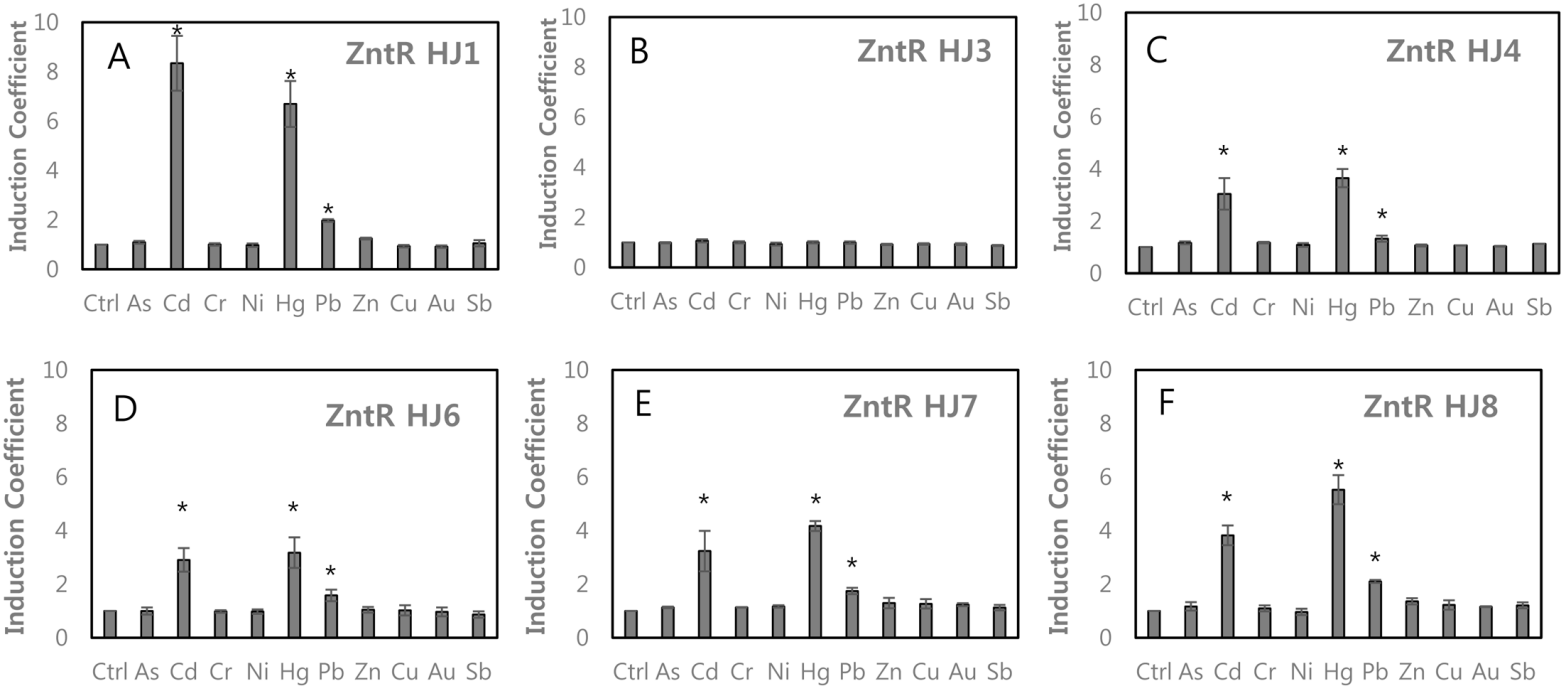
The metal(loid) selectivity of WCBs based on *E. coli*-*zntR* with ZntR possessing mutations on Cys115, H119 and Ser124. (**A**) ZntR-HJ1(His119Arg), (**B**) ZntR-HJ3 (Ser125Val), (**C**) ZntR-HJ4 (His119Arg/Ser125Val), (**D**) ZntRHJ6(Cys115Asp/His119Arg/Ser125Val), (**E**) ZntR-HJ7 (Cys115Ser/ His119Arg/Ser125Val), (**F**) ZntR-HJ8 (Cys115Glu/ His119Arg/Ser125Val). The asterisk (*) means the data were significantly higher than the control (*p* < 0.05).

**Effects of charged amino acids.** The responses of the first set of ZntR mutants with His119 and Ser125 mutations, denoted HJ1, HJ3, and HJ4, to various metal(loid) ions were evaluated (Fig. 2). The selectivity of HJ1 was similar and specificity was higher than those of ZntR-WT, as shown in Fig. 1. The responses toward metal(loid) ions were abolished by the Ser125Val mutation (ZntR-HJ3) and were restored by His119Arg/Ser125Val (ZntR-HJ4) (Figs. 2B and 2C). However, overall responses toward Cd and Hg decreased. These results clearly showed that the responses toward Pb were related to ZntR-HJ1 and HJ4 and responses toward Cd and Hg were decreased in HJ4.

**Effects of cysteines.** Although both ZntR-HJ1 and HJ4 showed responses to Pb, ZntR-HJ4 was selected as a template for further engineering to target Pb because of the decreased responses to Cd and Hg. Cys115 of ZntR-HJ4 was replaced with Asp, Ser, and Glu to generate another set of ZntR mutants, HJ6, 7, and 8, respectively. As shown in Figs. 2D–2F, the responses toward various metal(loid) ions were similar to those of HJ4. However, the responses to Pb increased slightly by replacing Cys115 with Asp, Ser, and Glu. In the case of ZntR-HJ8 (Cys115Glu/His119Arg/Ser125Val), the induction coefficient for Pb was greater than 2.0 (Fig. 2F). These results suggested that the mutation of Cys115 weakened the interactions of ZntR with Cd and Hg, the original ligands, and negatively charged residues promote the interaction of ZntR with other metal(loid) ions, such as Pb. Another key residue for metal ion sensing is Cys114, which is the first cysteine located on MBL. When it was replaced with Asp, Gly, Ser, and Pro from ZntR-WT to generate ZntR-HJ9 to 12, respectively, the metal(loid) specificities of WCBs were abolished, except for Cd and Hg (Fig. S1). However, the level of eGFP expression was low, with induction coefficients of < 2.0 for Cd and Hg. Accordingly, Cys114 was a pivotal residue in ZntR for interactions with metal(loid) ions.

**Fig. 3:**
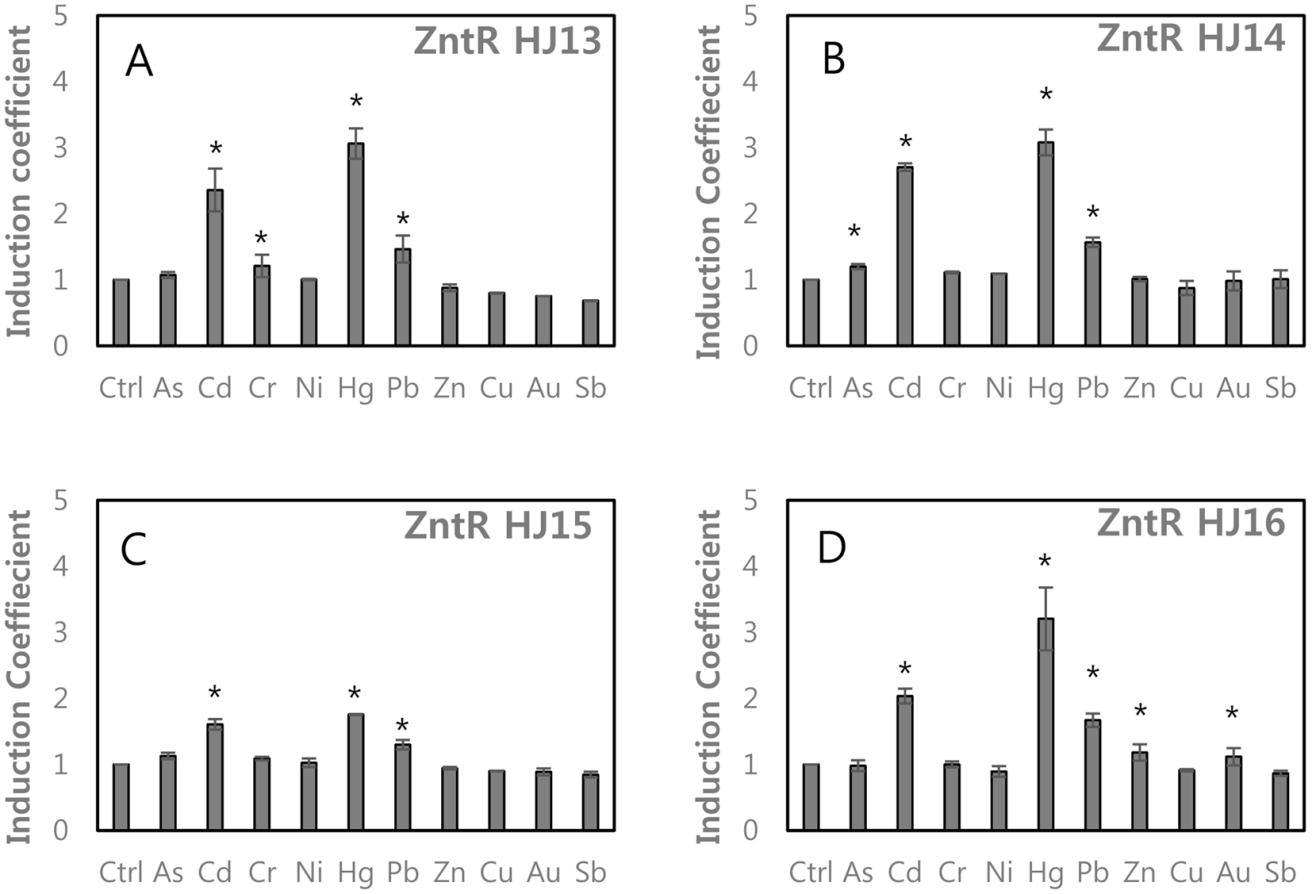
Alteration of metal(loid) selectivity of WCBs upon Cys115 mutations on ZntR-His119Arg/ Ser125Gly. (**A**) ZntR-HJ13 (His119Arg/Ser124Gly), (**B**) ZntR-HJ14 (Cys115Asp/ His119Arg/Ser124Gly), (**C**) ZntR-HJ15 (Cys115Ser/His119Arg/Ser124Gly), (**D**) ZntR-HJ16 (Cys115Glu/ His119Arg/Ser124Gly). The asterisk (*) means the data were significantly higher than the control (*p* < 0.05).

Since ZntR-HJ4, 6, 7, and 8 with Cys115, His119, and Ser125 maintained metal-sensing properties with slight differences from those of ZntR-WT, we used these to generate additional mutants. We predicted that the flexibility of MBLs affects the metal-sensing properties of ZntR. In this regard, Gly was replaced by Val125 in ZntR-HJ4, 6, 7, to 8 to generate ZntR-HJ13–16 (Fig. 3). ZntR-HJ13 (His119Arg/Ser125Gly) showed similar responses to HJ4 (His119Arg/Ser125Val) and the others also showed similar metal(loid) selectivity to HJ6, 7, and 8. Additionally, the responses to Pb were observed while responses to Cd and Hg were decreased (Figs. 3B-3D). The selectivity toward Pb was enhanced by Val125Gly from ZntR-HJ6, HJ7, and HJ8.

The His119Arg mutation had a positive effect on ZntR interactions with Pb, irrespective of changes in the induction coefficient. To verify the effect of His119Arg on Cys114 mutants (ZntR HJ9–12), ZntR-HJ17–20 were generated by replacing His119 with Arg. Interestingly, ZntR-HJ17 (Cys114Asp/His119Arg) and HJ20 (Cys114Pro/His119Arg) showed restored responses toward Cd and Hg (Fig. S2), while the others showed no response to any metal(loid) ions (data not shown). Compared to the metal(loid) responses of WCBs with ZntR-HJ9 and HJ12 (Figs. S1A and S1D), WCBs with ZntR-HJ17 and HJ20 harboring an additional His119Arg mutation showed responses toward Cd and Hg. Consequently, the metal(loid) specificity abolished by the Cys114 mutation was restored by an additional His119Arg mutation.

**Fig. 4:**
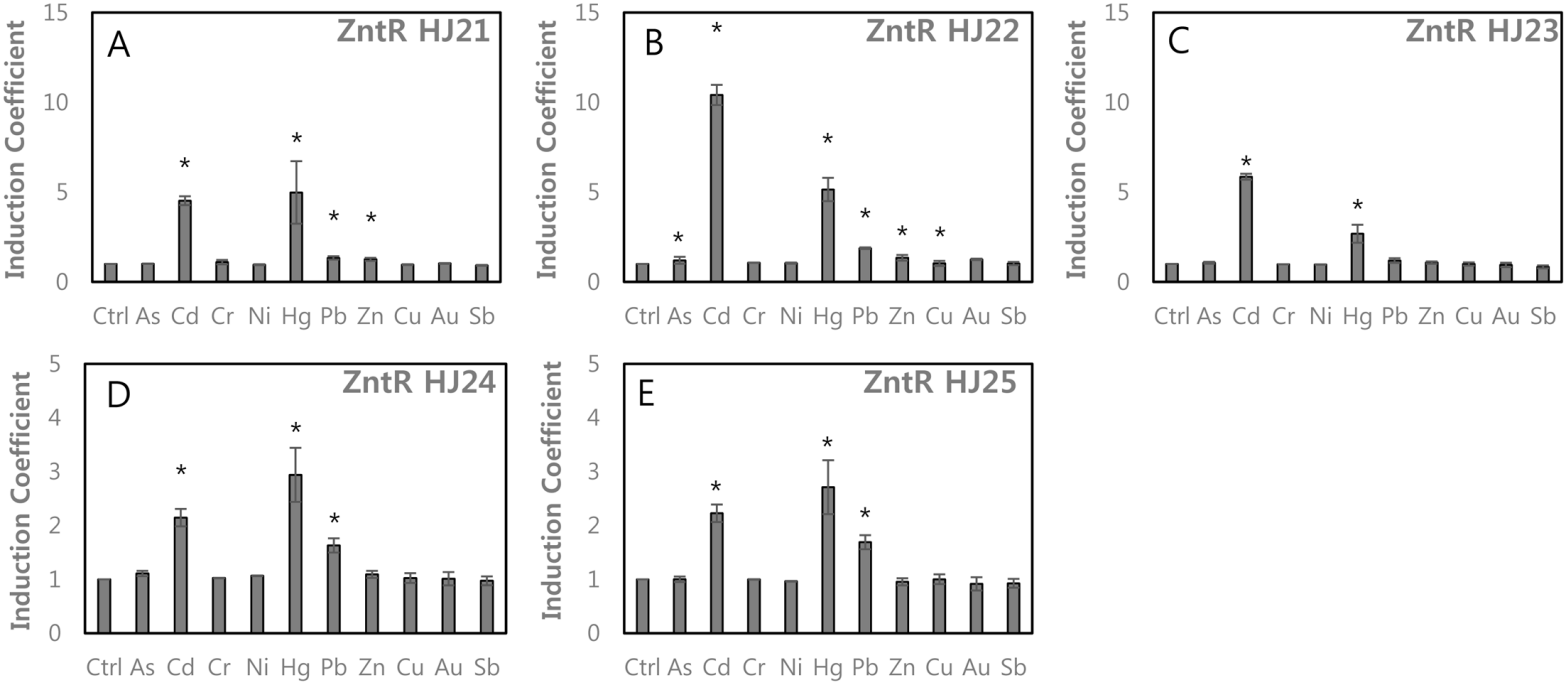
The effects of cysteine mutation with the insertion of charged residues on MBLs on metal(loid)s selectivity of WCBs. (**A**) ZntR-HJ21 (Gly116Asp/His119Arg), (**B**) ZntR-HJ22 (His119Arg/Val122Asp), (**C**) ZntR-HJ23 (Gly116Asp/His119Arg/Val122Asp), (**D**) ZntR-HJ24 (Cys115Asp/His119R/Ser120Asp/Ser125Gly), (**E**) ZntR-HJ25 (Cys115Glu/ His119R/Ser120Asp /Ser125Gly). The asterisk (*) means the data were significantly higher than the control (*p* < 0.05).

**Combined effects of charged amino acids and cysteines.** Our results indicated that His119Arg and the replacement of Cys115 with charged residues on ZntR altered the metal-sensing properties of WCBs. Accordingly, a set of ZntR loci possessing mutations at Cys115, G116, His119, Val122, and Ser125 were generated and are denoted ZntR-HJ21–HJ25 (Table 1). All 5 WCBs showed responses to Cd and Hg with various induction coefficients (Fig. 4). The WCB with ZntR-HJ21 (Gly116Asp/His119Arg) showed weaker responses to Cd and Hg than those of ZntR-HJ1 (His119Arg) (Fig. 4A). Gly116Asp weakened the specificity of ZntR to Cd and Hg. The WCBs with ZntR-HJ22 (His119Arg/Val122Asp) and HJ23 (Gly116Asp/His119Arg/ Val122Asp) showed similar selectivity toward metal(loid) ions to that of ZntR-HJ1 (His119Arg) (Figs. 4B and 4C). Based on these results, the addition of negatively charged residues, such as Asp, influenced the metal(loid) interactions. Two additional mutants, denoted ZntR-HJ24 and HJ25, were generated from ZntR-HJ14 and HJ16 by adding a negatively charged residue, Asp, to Ser120. Relative selectivity to Pb was enhanced because the overall responses to Cd and Hg were decreased (Figs. 4D and 4E).

### Effects of Interfering Metal Homeostasis Systems

**Fig. 5:**
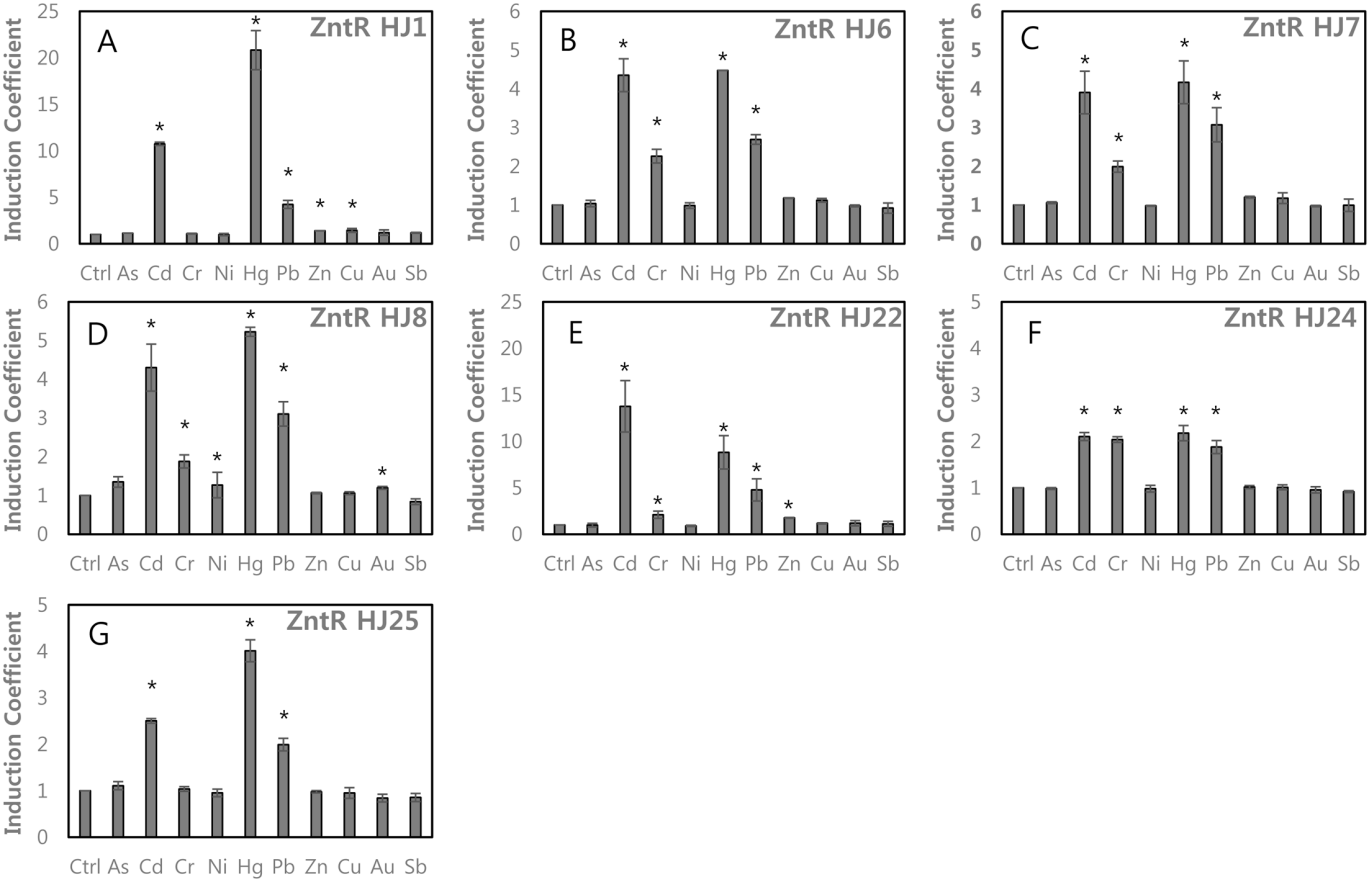
The effects of deleting *copA* on metal(loid) selectivity of WCBs. The *copA* deleted *E. coli*-*zntR*/*copA* was used as host stains for WCBs harboring pZnt-eGFP and ZntR-mutants. (**A**) ZntR-HJ1, (**B**) ZntR-HJ6, (**C**) ZntR-HJ7, (**D**) ZntR-HJ8, (**E**) ZntR-HJ22, (**F**) ZntR-HJ24, (**G**) ZntR-HJ25. The asterisk (*) means the data were significantly higher than the control (*p* < 0.05).

*E. coli* DH5α-*zntR* (*copA::kan*) was used as a host strain to test the effects of disrupting metal homeostasis on WCBs. Since *copA* encodes CopA, which is involved in the export of endogenous metal ions including copper, it plays a role in maintaining metal homeostasis in *E. coli* [16, 17]. Thus, the absence of CopA is expected to induce the accumulation of certain metal ions in cells, thereby enhancing the responses to metal ions. Based on the results obtained above, ZntR WT and derivatives, including HJ1, HJ6, HJ7, HJ8, HJ22, HJ24, and HJ25, were introduced into *E. coli* DH5α-*zntR*/*copA* with pZnt-eGFP to obtain WCBs. The metal(loid) selectivity of WCBs was tested in the same experimental conditions used in previous analyses. As shown in Fig. 5, the overall responses of WCBs were greater than those of the WCBs generated from *E. coli* DH5α-*zntR*, as expected. In the case of HJ1 and HJ22, the responses to Cd increased by 1.3-and 1.4-fold and the responses to Hg increased 3.0- and 1.8-fold. Additionally, the responses to Pb increased, with induction coefficients of 4.2 and 4.8, respectively (Figs. 5A and 5E). Others also showed greater signal intensities and some showed enhanced selectivity to Pb (Fig. 5). Although WCBs with HJ1 and HJ22 showed the best responses toward Pb based on induction coefficients, they were insufficient for Pb sensing due to the strong responses to Cd and Hg. The responses to Pb were relative to Hg or Cd for HJ7 and HJ24 were 0.73 and 0.86, respectively, while those for HJ1 and HJ22 were 0.25 and 0.35, respectively (Figs. 5C and 5F). Notably, a response toward Cr was newly observed with induction coefficients >2.0, although the signals were not significant (Fig. 5). However, the relative response to Cr was similar to those to other metal(loid)s, such as Cd, Hg, and Pb, in case of ZntR-HJ24 (Fig. 5F).

## Discussion

WCBs are promising alternatives for the quantification of environmental pollutants owing to their simplicity, rapidity, and low cost [10, 18, 19]. WCBs share a sensing domain and reporter domains that recognize and indicate the presence of target contaminants, respectively. Sensing domains repress reporter gene expression by binding to the promoter region without targets. Interaction with targets induces a conformational change to recruit RNA polymerase to the promoter region to initiate the transcription of the reporter gene [20, 21]. Thus, targets are easily quantified by measuring the expression level of reporter genes. Owing to this simple process, WCBs have been actively investigated in the past few decades. However, the number of genetic systems that could be used for WCBs were relatively limited to cover the environmental toxicants and pollutants, even though a large amount of genetic data on organisms has been accumulated these days. In this regard, it is important to modulate the sensitivity and selectivity of available WCBs by genetic engineering as well as to find out new genetic systems. In this study, we demonstrated the generation of WCBs with new targets by modulating the binding properties of sensing domains regarded as regulatory proteins in stress-inducible operons along with interfering with the function of genes involved in homeostasis systems in bacterial cells.

ZntR, a metalloregulator in the zinc-responsive operon in *E. coli*, senses Cd, Hg, and Zn and is used for bacterial cell-based biosensors to monitor those metal(loid) ions [22, 23]. Thus, WCBs employing ZntR could not be used to monitor specific target metal(loid) ions owing to the broad specificity of this regulator protein [24]. In fact, the attempts to modulate the properties of WCBs by introducing mutation on ZntR had been reported [15]. Even though studies were performed under different experimental conditions and systems, they showed the mutations on ZntR were able to change the properties. However, the metal selectivity of WCBs was presumed to Cd and Zn while the level of specificity was modulated. In concordance with this study, the new metal(loid)-sensing WCBs were not obtained by mutating the MBL region of ZntR in our previous studies, while single metal(loid) ion-specific WCBs (i.e., Cd-specific and Hg-specific WCBs) were obtained [5, 7]. Nonetheless, we thought the novel targets of WCBs could be obtained if the regulatory proteins possess the ability to interact with novel targets. To validate the idea, the mutations on the metal-binding site of ZntR were selected from the amino acid sequence alignment analysis with other metalloregulatory proteins.

PbrR691 is a Pb-binding metalloregulatory protein [14, 25, 26]. It belongs to the Mer family of proteins and interacts specifically with Pb ions. A pyramid is formed from the Pb ion and 3 cysteines in PbrR 691. Additionally, Arg117 and Gly123 in MBL of PbrR691 stabilize the interaction with Pb ions by forming a binding pocket and van der Waals interactions. We replaced His119 and Ser125 in MBL of ZntR with Arg and Gly/Val, respectively, and introduced additional mutations on Cys78, Cys114 and Cys124 known as key residues; they form a structural pocket for interactions with metal ions [13, 15]. In the case of ZntR-HJ1 (His119Arg), WCBs showed about 2-fold increased signals, when compared with that for the WT, and showed a slightly increased response to Pb (Fig. 2A). In the case of ZntR-HJ4, the responses toward Cd and Hg were decreased by the Ser125Val mutation. It was difficult to explain why the metal-binding properties were changed by these mutations, but it is possible that the structural coordination of MBLs and metal(loid) ions was affected by point mutations.

When Cys115 was replaced with Asp, Ser, and Glu, responses toward Pb increased. In particular, responses toward Pb were 2.2 and 1.5 times greater than those of ZntR-WT with Cys115Glu including ZntR-HJ8 and HJ16 (Figs. 3C and 4D). Interestingly, although mutations at Cys114 abolished metal(loid) responses (Fig. S1), the responses were restored by Cys114Asp and Cys114Pro with His119Arg (Fig. 5). Additionally, negatively charged residues, such as Asp and Glu, were introduced on MBL regions with His119Arg. The resulting ZntR-HJ21–HJ25 still showed Cd and Hg specificity but replacing Cys115 with other residues weakened the responses toward Cd and Hg, thereby increasing the specificity toward Pb (Figs. 4D and 4E).

ZntR mutants with Pb responses, such as HJ1, HJ6, HJ7, HJ8, HJ22, HJ24, and HJ25, were also introduced into *E. coli*-*zntR*/*copA* (Δ*zntR*/*copA::kan*) strains to evaluate the effects of copA, known to export copper ions out of cells [16, 27]. *copA* is classified into the P-type ATPase family, and P1B-type ATPases are known to transport Cu(I), Cu(II), Zn(II), Cd(II), Co(II), and Pb(II) across cell membranes [28]. Thus, we expected the responses toward metal(loid) ions to increase by deleting copA. The responses of WCBs to Pb were around 2 to 5 times greater; interestingly, increased responses toward Cr were observed (Figs. 5 and S2). Although speculative, our results suggest that CopA in *E. coli* is related to regulating the concentrations of Cr and Pb as well as Cu. Additionally, some of ZntR mutants showed new responses to Cr and Pb. Although they did not specifically respond to single metal ions, it would be possible to detect Pb or Cr in environmental systems. To validate the ability of the newly obtained WCBs to quantitatively detect Pb and Cr, we evaluated WCBs possessing ZntR-HJ7 and HJ24, respectively (Fig. S3). The responses of WCBs increased in concentration-dependent manners. Although we still observed responses toward Cd and Hg, these WCBs could be used for Cr and Pb sensing. Of course, it is true that using other genetic systems to detect Cr and Pb and the HJ7 and HJ24 were not good enough to sense those ions. Nonetheless, it would be valuable to generate Cr- and Pb-sensing WCBs from *znt*-operon system. In conclusion, we believed it would be important to show the possibility of generating new metal(loid)-sensing WCBs from existing WCB systems, while also demonstrating that this strategy has the potential to substantially expand the number of targets and corresponding WCBs.

## Supplemental Materials



Supplementary data for this paper are available on-line only at http://jmb.or.kr.

## Figures and Tables

**Table 1:** The list of recombinant ZntR wild type and mutants tested in this study with amino acid sequences of metal-binding loops (MBLs).

No.	Name	Amino acid sequences of MLBs	Order of responding strength	

			*E. coli*-*zntR*	*E. coli*-*zntR*/*copA*
1	ZntR-WT	CCGTAHSSVYCS	Cd>Hg	Cd>Hg
2	ZntR-HJ1	CCGTA**R**SSVYCS	Cd>Hg>Pb	Hg>Cd>Pb
3	ZntR-HJ3	CCGTAHSSVYC**V**	None	-
4	ZntR-HJ4	CCGTA**R**SSVYC**V**	Hg>Cd	-
5	ZntR-HJ6	C**D**GTA**R**SSVYC**V**	Hg>Cd	Hg=Cd>Pb>Cr
6	ZntR-HJ7	C**S**GTA**R**SSVYC**V**	Hg>Cd>Pb	Hg=Cd>Pb>Cr
7	ZntR-HJ8	C**E**GTA**R**SSVYC**V**	Hg>Cd>Pb	Hg>Cd>Pb>Cr
8	ZntR-HJ9	**D**CGTAHSSVYCS	None	-
9	ZntR-HJ10	**G**CGTAHSSVYCS	None	-
10	ZntR-HJ11	**S**CGTAHSSVYCS	None	-
11	ZntR-HJ12	**P**CGTAHSSVYCS	None	-
12	ZntR-HJ13	CCGTA**R**SSVYC**G**	Hg>Cd	-
13	ZntR-HJ14	C**D**GTA**R**SSVYC**G**	None	-
14	ZntR-HJ15	C**S**GTA**R**SSVYC**G**	Hg>Cd	-
15	ZntR-HJ16	C**E**GTA**R**SSVYC**G**	Hg>Cd >Pb	-
16	ZntR-HJ17	**D**CGTA**R**SSVYCS	Hg>Cd	-
17	ZntR-HJ18	**G**CGTA**R**SSVYCS	None	-
18	ZntR-HJ19	**S**CGTA**R**SSVYCS	None	-
19	ZntR-HJ20	**P**CGTA**R**SSVYCS	Hg>Cd	-
20	ZntR-HJ21	CC**D**TA**R**SSVYCS	Hg>Cd	-
21	ZntR-HJ22	CCGTA**R**SS**D**YCS	Cd>Hg>Pb	Cd>Hg>Pb>Cr
22	ZntR-HJ23	CC**D**TA**R**SS**D**YCS	Cd>Hg	-
23	ZntR-HJ24	C**D**GTA**RD**SVYC**G**	Hg>Cd >Pb	Cd=Hg=Pb=Cr
24	ZntR-HJ25	C**E**GTA**RD**SVYC**G**	Hg>Cd >Pb	Hg>Cd >Pb

^1^The metal(loid) ions inducing over 2.0 of induction coefficient values were included, and the WCBs showing less than 2 of induction coefficient values were excluded for further tests.

^2^*E. coli*-*zntR* and *E. coli*-*zntR*/*copA* represented the genetically engineered *E. coli* strains with Δ*zntR::kan* and Δ*zntR*/*copA::kan*, respectively.

^3^The bold and underlined letters indicated the point mutations on MBLs.

## References

[1] Bereza-Malcolm LT, Mann Gl, Franks AE (2014). Environmental sensing of heavy metals through whole cell microbial biosensors: a synthetic biology approach. ACS Synthetic Biol..

[2] Fernandez-López R, Ruiz R, de la Cruz F, Moncalián G (2015). Transcription factor-based biosensors enlightened by the analyte. Front. Microbiol..

[3] Mahr R, Frunzke J (2016). Transcription factor-based biosensors in biotechnology: current state and future prospects. Appl. Microbiol. Bbiotechnol..

[4] Robbens J, Dardenne F, Devriese L, De Coen W, Blust R (2010). *Escherichia coli* as a bioreporter in ecotoxicology. Appl. Microbiol. Bbiotechnol..

[5] Yoon Y, Kang Y, Lee W, Oh KC, Jang G, Kim BG (2018). Modulating the properties of metal-sensing whole-cell bioreporters by interfering with *Escherichia coli* metal homeostasis. J. Microbiol.Biotechnol..

[6] Kang Y, Lee W, Kim S, Jang G, Kim BG, Yoon Y (2018). Enhancing the copper-sensing capability of *Escherichia coli*-based whole-cell bioreporters by genetic engineering. Appl. Microbiol. Bbiotechnol..

[7] Kang Y, Lee W, Jang G, Kim BG, Yoon Y (2018). Modulating the sensing properties of *Escherichia coli*-based bioreporters for cadmium and mercury. Appl. Microbiol. Bbiotechnol..

[8] Yoon Y, Kim S, Chae Y, Kang Y, Lee Y, Jeong SW (2016). Use of tunable whole-cell bioreporters to assess bioavailable cadmium and remediation performance in soils. PLoS One.

[9] Guo M, Du R, Xie Z, He X, Huang K, Luo Y, Xu W (2019). Using the promoters of MerR family proteins as "Rheostats" to engineer whole-cell heavy metal biosensors with adjustable sensitivity. J. Biol. Eng..

[10] Xu T, Close DM, Sayler GS, Ripp S (2013). Genetically modified whole-cell bioreporters for environmental assessment. Ecol. Indic..

[11] Cerminati S, Soncini FC, Checa SK (2015). A sensitive whole-cell biosensor for the simultaneous detection of a broad-spectrum of toxic heavy metal ions. Chem. Commun..

[12] Ibáñez MM, Checa SK, Soncini FC (2015). A single serine residue determines selectivity to monovalent metal ions in metalloregulators of the MerR family. J. Bacteriol..

[13] Changela A, Chen K, Xue Y, Holschen J, Outten CE, O'Halloran TV (2003). Molecular basis of metal-ion selectivity and zeptomolar sensitivity by CueR. Science.

[14] Huang S, Liu X, Wang D, Chen W, Hu Q, Wei T (2016). Structural basis for the selective Pb (II) recognition of metalloregulatory protein PbrR691. Inorg. Chem..

[15] Khan S, Brocklehurst KR, Jones GW, Morby AP (2002). The functional analysis of directed amino-acid alterations in ZntR from *Escherichia coli*. Biochem. Biophys. Res. Commun..

[16] Rensing C, Fan B, Sharma R, Mitra B, Rosen BP (2000). CopA: an *Escherichia coli* Cu (I)-translocating P-type ATPase. Proc. Natl Acad. Sci..

[17] Stoyanov JV, Hobman JL, Brown NL (2001). CueR (YbbI) of *Escherichia coli* is a MerR family regulator controlling expression of the copper exporter CopA. Mol. Microbiol..

[18] Belkin S (2003). Microbial whole-cell sensing systems of environmental pollutants.

[19] Harms H, Wells MC, van der Meer JR (2006). Whole-cell living biosensors?re they ready for environmental application? Appl. Microbiol. Biotechnol..

[20] Leveau JH, Lindow SE (2002). Bioreporters in microbial ecology. Curr. Opin. Microbiol..

[21] Wang B, Barahona M, Buck M (2013). A modular cell-based biosensor using engineered genetic logic circuits to detect and integrate multiple environmental signals. Biosens. Bioelectron..

[22] Magrisso S, Erel Y, Belkin S (2008). Microbial reporters of metal bioavailability. Microb. Biotechnol..

[23] Brocklehurst KR, Hobman JL, Lawley B, Blank L, Marshall SJ, Brown NL (1999). ZntR is a Zn (II)‐responsive MerR‐like transcriptional regulator of zntA in *Escherichia coli*. Mol. Microbiol..

[24] Gireesh-Babu P, Chaudhari A (2012). Development of a broad-spectrum fluorescent heavy metal bacterial biosensor. Mol. Biol. Rep..

[25] Chen PR, Wasinger EC, Zhao J, Van Der Lelie D, Chen LX, He C (2007). Spectroscopic insights into lead (II) coordination by the selective lead (II)-binding protein PbrR691. J. Am. Chem. Soc..

[26] Chen P, Greenberg B, Taghavi S, Romano C, van der Lelie D, He C (2005). An exceptionally selective lead (II)‐regulatory protein from ralstonia metallidurans: Development of a fluorescent lead (II) Probe. Angew. Chem. Int. Ed. Engl..

[27] Petersen C, Møller LB (2000). Control of copper homeostasis in *Escherichia coli* by a P-type ATPase, CopA, and a MerR-like transcriptional activator, CopR. Gene.

[28] Argüello JM, Eren E, González-Guerrero M (2007). The structure and function of heavy metal transport P 1B-ATPases. Biometals.

