# Supplementary material for: Modulation of the Metal(loid) Specificity of Whole-Cell Bioreporters by Genetic Engineering of ZntR Metal-Binding Loops
Source: J Microbiol Biotechnol. 2020 Feb 10;30(5):681–8. doi: 10.4014/jmb.1911.11053 (PMC9728388; doi:10.4014/jmb.1911.11053)

J. Microbiol. Biotechnol.
https://doi.org/10.4014/jmb.1911.11053

Supplementary data for:

ulation of the metal(loid) specificity of whole-cell bioreporters by the genetic engineering of ZntR metal-binding loops

Hyojin Kim[1], Geupil Jang[2], Bong-Gyu Kim[3], Youngdae Yoon[1,*]

[1]*Department of Environmental Health Science, Konkuk University, Seoul 05029, Republic of Korea*
[2]*School of Biological Sciences and Technology, Chonnam National University, Gwangju 61186, Republic of Korea*
[3]*Department of Forest Resources, Gyeongnam National University of Science and Technology, Jinju 52725, Republic of Korea*

[*]Correspondence to: Youngdae Yoon
Tel: +82-2-450-0443
Fax: +82-2-450-3726
E-mail: yyoon21@gmail.com

Figure S1. Effect of Cys114 mutagenesis of ZntR on metal(loid) specificity of WCBs. The experiments were repeated more than 3 times and the error bars were represented as the standard deviations. The asterisk (*) means the data was significantly higher than the control ($p < 0.05$).

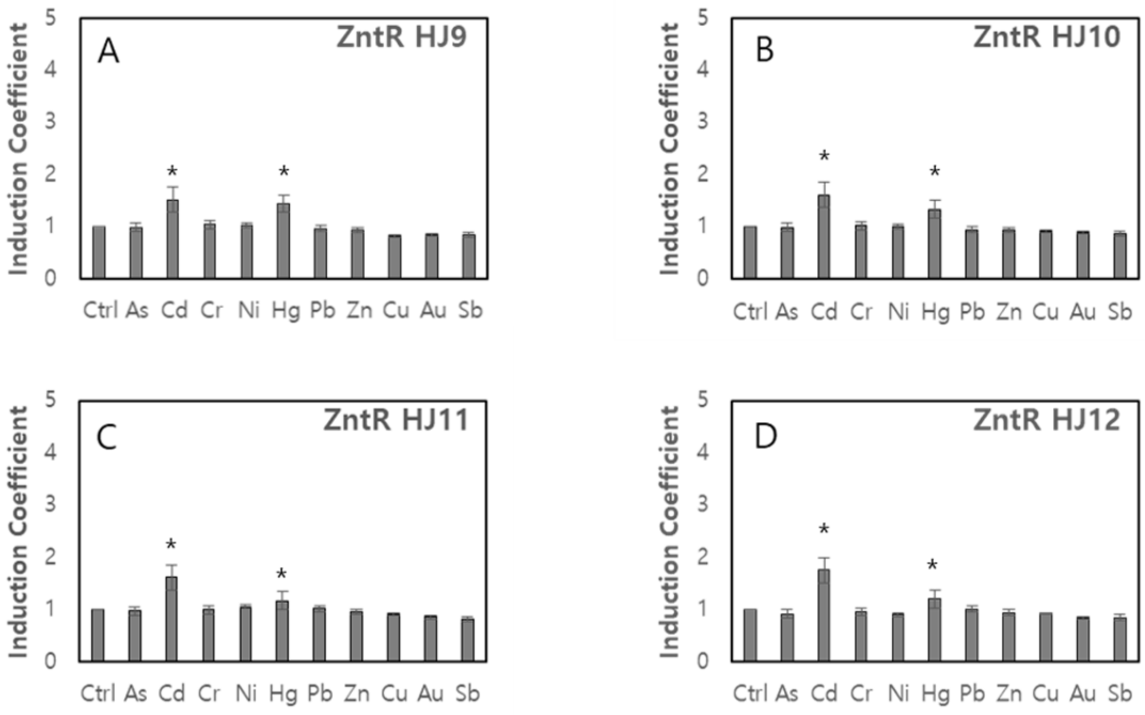

Figure S2. Effect of Cys114 mutagenesis of ZntR on metal(loid) specificity of WCBs based on *E. coli-zntR.* The experiments were repeated more than 3 times and the error bars were represented as the standard deviations. The asterisk (*) means the data was significantly higher than the control ($p < 0.05$).

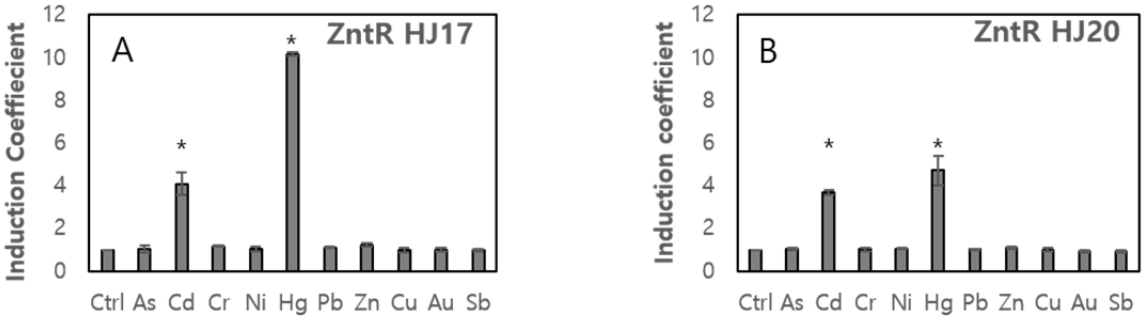

Figure S3. Standard curves for quantifying Pb and Cr using WCBs based on *E. coli-zntR/copA* harboring ZntR-HJ7 and ZntR-HJ24, respectively. The experiments were repeated more than 3 times and the error bars were represented as the standard deviations.

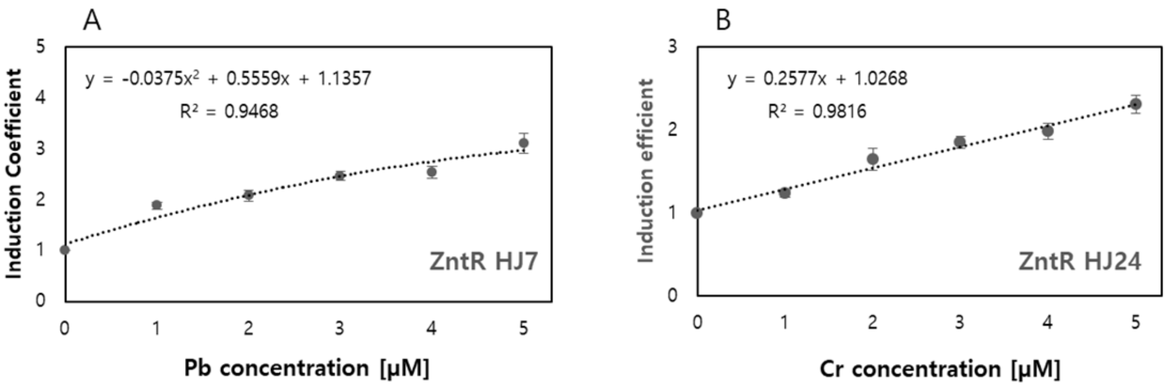

Supplement: Supplementary file 1 [file JMB-30-5-681-supple.pdf]
